# Quantum and Electromagnetic Fields in Our Universe and Brain: A New Perspective to Comprehend Brain Function

**DOI:** 10.3390/brainsci11050558

**Published:** 2021-04-28

**Authors:** Zamzuri Idris, Zaitun Zakaria, Ang Song Yee, Diana Noma Fitzrol, Abdul Rahman Izaini Ghani, Jafri Malin Abdullah, Wan Mohd Nazaruddin Wan Hassan, Mohd Hasyizan Hassan, Asrulnizam Abdul Manaf, Raymond Ooi Chong Heng

**Affiliations:** 1 Department of Neurosciences, School of Medical Sciences, Universiti Sains Malaysia, Kubang Kerian 16150, Malaysia; zakariaz@tcd.ie (Z.Z.); happy_4428@hotmail.com (A.S.Y.); diana_noma@hotmail.com (D.N.F.); yoppghani@gmail.com (A.R.I.G.); brainsciences@gmail.com (J.M.A.); 2 Brain and Behaviour Cluster (BBC), School of Medical Sciences, Universiti Sains Malaysia, Kubang Kerian 16150, Malaysia; 3 Hospital Universiti Sains Malaysia (HUSM), Universiti Sains Malaysia, Kubang Kerian 16150, Malaysia; drnaza_anaest@yahoo.co.uk (W.M.N.W.H.); hasyizan@usm.my (M.H.H.); 4 Department of Anaesthesiology, School of Medical Sciences, Universiti Sains Malaysia, Kubang Kerian 16150, Malaysia; 5 Collaborative Microelectronic Design Excellence Center (CEDEC), Universiti Sains Malaysia, Bayan Lepas 11900, Malaysia; eeasrulnizam@usm.my; 6 Department of Physics, Faculty of Science, University of Malaya, Kuala Lumpur 50603, Malaysia; rooi@um.edu.my

**Keywords:** brainwaves, electromagnetic field, quantum field, consciousness, quantum mechanics, Bohmian mechanics

## Abstract

The concept of wholeness or oneness refers to not only humans, but also all of creation. Similarly, consciousness may not wholly exist inside the human brain. One consciousness could permeate the whole universe as limitless energy; thus, human consciousness can be regarded as limited or partial in character. According to the limited consciousness concept, humans perceive projected waves or wave-vortices as a waveless item. Therefore, human limited consciousness collapses the wave function or energy of particles; accordingly, we are only able to perceive them as particles. With this “limited concept”, the wave-vortex or wave movement comes into review, which also seems to have a limited concept, i.e., the limited projected wave concept. Notably, this wave-vortex seems to embrace photonic light, as well as electricity and anything in between them, which gives a sense of dimension to our brain. These elements of limited projected wave-vortex and limitless energy (consciousness) may coexist inside our brain as electric (directional pilot wave) and quantum (diffused oneness of waves) brainwaves, respectively, with both of them giving rise to one brain field. Abnormality in either the electrical or the quantum field or their fusion may lead to abnormal brain function.

## 1. Introduction

Our universe and brain obviously require energy. Energy is described in many ways and comprises chemical, metabolic, radiation, and many other types. In physics, energy has been noted as having one peculiar feature—ups and downs. This wavy form of energy is thought of as covering our entire universe, including our body and brain. This idea came about after realizing that an atom or light can exist in two forms, both as a particle and as a wave [1,2,3,4,5,6]. This is known as the duality of an atom or light. Therefore, we can also view all things in our universe as waves. In such a way, our brain can be viewed at the deepest level as either an ensemble of particles or a wavy brain. Hence, a picture of the wavy brain should at least have two components of waves: electromagnetic brainwaves and waves arising from all particles (the anatomical chemical component or mass itself is an energy). Many think that these whole waves are not only restricted to the brain, but that they easily diffuse out and form an interconnected wave network with our universe. This notion is accepted by some neuroscientists and physicists, which is associated with consciousness [7,8,9] or string theory [10,11].

**Figure 1:**
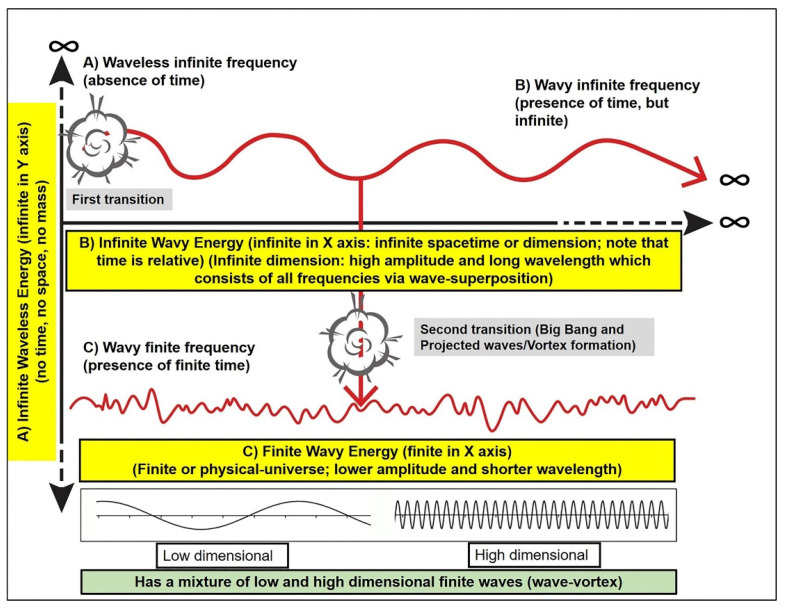
The two transitional points of the universe. The first transition is at the point between waveless (**A**) and wavy infinite energy (**B**). The second transition (the singularity or origin of our universe) is at the point between wavy infinite energy (**B**) and wavy finite energy (**C**), which has a mixture of various wave patterns—low, high, and intermediate dimensional waves. Thus, infinite energy, infinite light, or infinite frequency (the highest light and all light) may have already existed before the singularity, and the origin of lower light could have been from higher light.

In 2019, the present corresponding author published a manuscript that described waveless energy [12]. Waveless energy is regarded as infinite energy or infinite light. The reason for it being waveless is because of its dissociation from time and space (dimension) (thus having infinite frequency); therefore, it was thought of as something that has existed since before the formation of our universe or singularity (commonly known as the Big Bang). Here, in this manuscript, the authors expand this idea by introducing another infinite energy, which is wavy in nature and related to infinite time and space (infinite wavy energy). This hypothetical energy can be viewed as lying in between infinite waveless energy and finite wavy energy. In other words, the newly introduced infinite wavy energy of the cosmos in this manuscript is in fact equivalent to the cosmos quantum field (QF), which is well established in string theory. The authors use both terms in order to make the readers better understand the concept of oneness or one consciousness (related to QF) and interception of energy (projected waves). Figure 1 illustrates these points and the transitions that might occur among infinite waveless, infinite wavy, and finite wavy energy. 

According to the aforementioned concept of these three energies (infinite waveless, infinite wavy, and finite wavy energy), the authors discuss and explain (a) the possible relationship that might exist between waveless and wavy energy in our universe and brain, and (b) the projected wave concept (vortex), which could explain (1) the duality of an atom, (2) our borderless universe, (3) the absence of wave interference in our three-dimensional (3D) physical universe, (4) the infinite shape (geometric pattern that is technically repeatable or limitless) of materials in our brain and nature, and (5) the existence of two energy fields (electromagnetic and quantum fields) inside our universe, as well as inside our brain, which could explain the pathogenesis of neurological (particularly psychiatric) disorders. Through this perspective, the authors hope that future directions in research will focus on quantum field and quantum energy detectors for the brain, which may further enhance our understanding of brain function, thereby facilitating the cure of more diseases.

## 2. Infinite and Finite Field Energy

Energy conservation is a principle in physics. There is no loss of energy; it is only converted to different forms. From the time of singularity, the total energy has been maintained but in different forms. Previously, we explained the possible existence of infinite light, frequency, or energy prior to the Big Bang [12]. This light is infinite because it is not related to time or space, and it is waveless. Thus, this sort of energy is considered waveless energy, which is difficult or impossible to detect simply because it is waveless. On the contrary, our newly introduced infinite wavy energy (or the cosmos quantum field as stated in string theory) is detectable and can be studied. To understand the cosmos QF or infinite wavy energy, one must consider the view of an atom from the perspective of quantum physics. According to quantum theory, an atom can exist in duality, i.e., either in particle or in wave form. Therefore, one may also perceive all things as waves or as energy. Then, the important question related to this perception is the following: “Where did this wavy energy originate from?” Since our initial statement maintains that energy preservation is always valid, then a hypothesis could be that this finite wavy energy originated from infinite wavy or infinite waveless energy; hence, both (infinite wavy and infinite waveless energy) are indeed infinite in their features.

Figure 1 also depicts the conservation of energy concept, whereby the *Y*-axis describing infinite waveless energy (with the absence of spacetime: “zero” spacetime) gives rise to another source of infinite energy, i.e., the *X*-axis describing infinite wavy energy with the presence of “infinite” spacetime or infinite dimension. Therefore, these two infinite energies are inter-related, and the total energy is always preserved: one in the vertical or *Y*-direction (in fact, in any direction) with waveless, timeless, spaceless, and limitless energy, and the other in the horizontal or *X*-axis infinite direction with the presence of spacetime or dimension. In addition, one may notice at least two possible transitional points in Figure 1: (a) between infinite waveless energy and infinite wavy energy, and (b) between infinite wavy energy and finite wavy energy. In short, one may view such a connection as infinite waveless–infinite wavy–finite wavy energy or, from an analogous perspective, as infinite light–infinite waves–finite particles/waves (duality).

**Figure 2:**
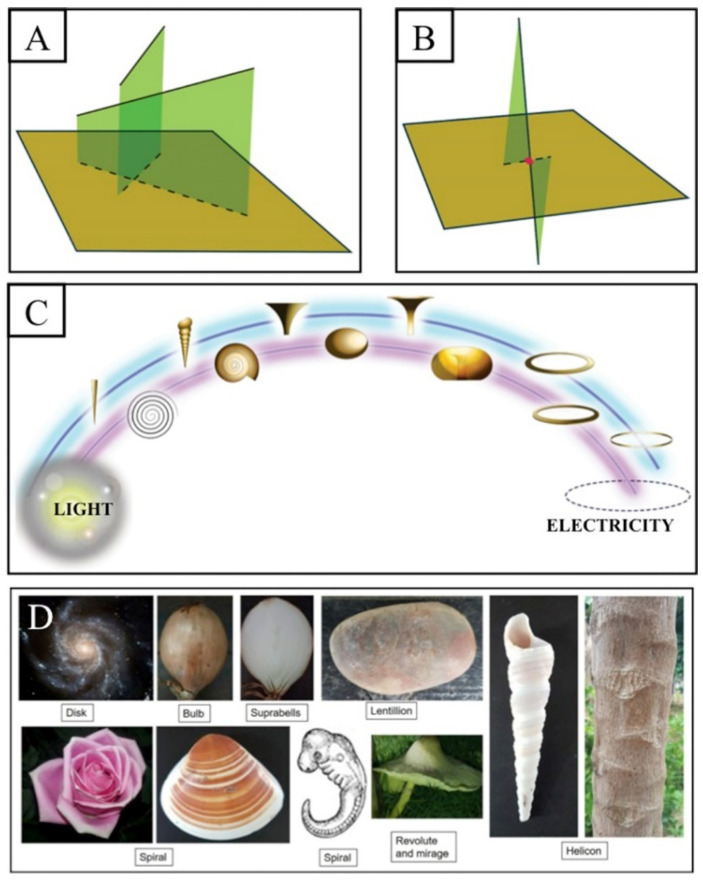
Limited projected waves and infinite shapes in the brain and nature. (**A**) Waves (green) in different dimensions. (**B**) Waves that really intersect our universe (a brown square) at the red point. (**C**) The intersected waves form wave-vortices that range from low- to high-dimensional waves (light to electricity vortices); matter and other vortices lie between these two vortices. (**D**) Limited projected waves give a sense of reality (which, in fact, describe the illusion of matter, collapsing its original wave or energy function), and their shapes (nature) are infinite shapes, denoting their infinite-wave origin. Interestingly, the brain also forms part of the projected waves and is indeed infinite in its shape; examples are the hippocampus as a logarithmic spiral, the caudate nucleus as a Dürer spiral, and the thalamus as a lemniscate.

**Figure 3:**
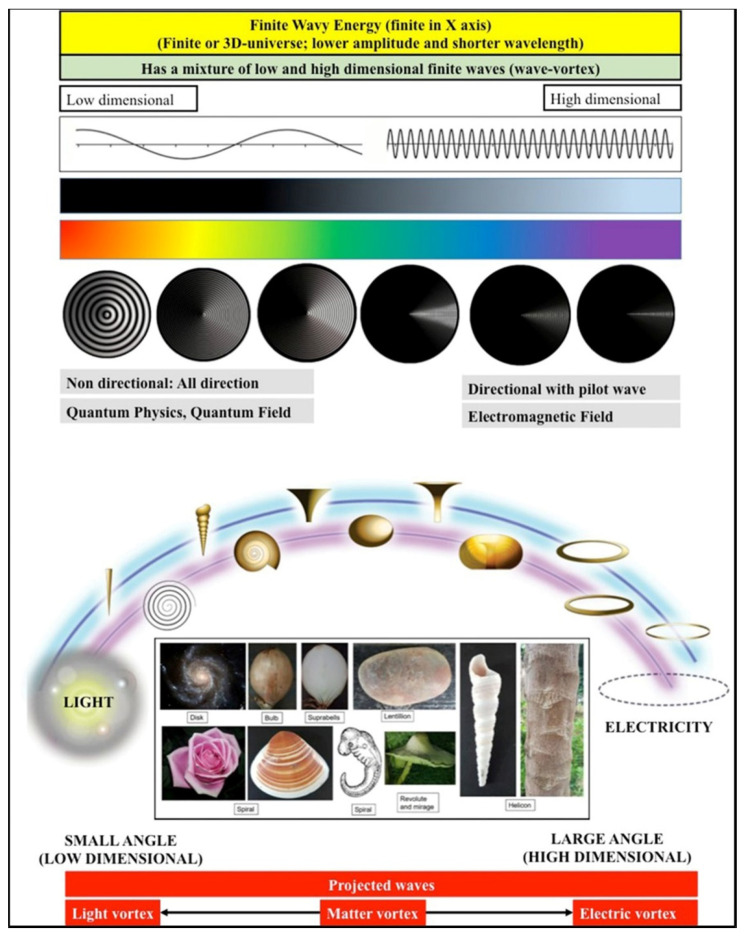
Illustration of further details on the low- and high-dimensional fields of our finite uni-verse. The intersection of higher and lower light creates light, electricity, and matter vortices. The light vortex is created by a small angle of interception, whereas electricity is created by a large angle of interception. Matter vortices are formed by the angles in between. Any light is regarded as low-dimensional and nondirectional, diffused, changing, or delocalized, whereas electricity is considered high-dimensional and directional with features of pilot waves, i.e., it is a locatable, deterministic, specific network that may follow Bohmian mechanics.

Then, another important question to answer is the following: “How was our physical universe formed?” To answer this, one should consider the second transitional point as illustrated in Figure 1 and return to the singularity. After the singularity, time (a new time period) began to exist (remember that time is only relative) and light was formed, followed by waves, particles, molecules, matter, and objects. The energy that was formed must again be wavy energy but finite along the *X*-axis (finite time and space). This wavy and finite energy formed our current 3D universe (finite dimensional universe). Thus, one may say that the wavy infinite energy which was initially derived from waveless infinite energy was the building stone for our physical or 3D universe. From a broader perspective, electromagnetic photonic light (photonic light vortex/light immediately after the singularity) was initially thought of as arising from an implosion and explosion (the origin of our universe from a singularity can also mean a transition without an actual loud bang) of this infinite wavy energy (which could also be a kind of light); subsequently, our 3D universe came into complete existence via projected waves of infinite wavy energy that ‘intersected’ the initially formed electromagnetic photonic light to form electricity and matter (Figure 1, Figure 2 and Figure 3). The word “vortex” used in this manuscript refers to “projected wave movement” (normally, it is referred to as rotational fluid or air movement). Further details are provided in Section 4.

## 3. Physical Universe and Mind (Observer)

Before proceeding to a vortex or projected wave, perhaps it is better to briefly examine the mind (observer). One important question related to the mind is the following: “Does our physical universe really exist?” Because of the peculiar features of our brain, this sort of question is commonly asked by many scientists [13,14]. We acknowledge that all the individual features of the world are experienced through our body sense organs. The information that reaches us through these organs is converted into electrical signals in the brain. In summary, our brains constitute only electrical signals. This causes many to question the nature of the perceiver. The perceiver is thought of by some as the mind [15,16]. Despite the absence of an electrical converter inside the brain, the mind can interpret and make these electrical signals meaningful. Some authors regard the mind as a spirit or soul (infinite item, timeless, spaceless) [12,16]. It is worthy to note that, despite the absence of external stimuli, such as when a person is dreaming, undergoing hypnosis, or experiencing virtual reality, the brain’s electrical signals (waves) are also activated. This is due to persistent biochemical activities, not only inside our brain but also inside the nerves around our body [17,18]. These represent possible proof that the brain does not necessarily require the material world to function. According to quantum theory and string theory, our physical world basically consists only of waves and vibrations. This is indeed in agreement with the quantum perspective of our brain, whereby only waves are present inside our brain and the acts of seeing, hearing, planning, etc., possibly happen via either entanglement or interconnected flow of wavy energy. This gives rise to yet another important question: “If so, why can the mind see matter, light, objects, etc., but not waves?” Indeed, waves and energy are subtle or abstract to the brain in that they cannot be seen by the normal brain. The reason for this might be related to the wave-vortex, the limited frequency for seeing, hearing, touching, etc., or the limitation of our human consciousness (i.e., having limited consciousness).

## 4. Wave and Consciousness as a Limitless (Infinite) and Limited (Finite) Entity

The aforementioned discussion stated that all things can apparently be described as waves (wavy energy), which originate from infinite wavy energy or, on a deeper level, from infinite waveless energy or an infinite light. This infinite wavy energy is limitless on a single *X*-axis (limitless or infinite in spacetime or dimension), whilst infinite waveless energy has infinite energy in any (infinite) direction. It is represented here as the *Y*-axis in Figure 1 due to the two-dimensional (2D) representation; thus, it has no time or space, but has infinite energy. Therefore, it appears that all things discussed here can be thought of as an infinite entity. This wholeness of waves, i.e., infinite waveless energy (infinite light) and infinite wavy energy (infinite waves, infinite dimension, energy, or all light), can be regarded as one consciousness, simply because they are inter-related and they give energy (life, existence, form) to all [14,19,20,21].

On the basis of the above argument, is it possible then that our 3D physical universe is not limitless, but instead a ‘limited or finite physical universe’? The reason for saying this is because of the limited wave-projection concept (i.e., finite waves are formed from a portion of projected infinite waves, referred to as vortices in this manuscript). Since all things can be regarded as waves, then perhaps one should also wonder why all these wave signals do not bump into each other. The reason that these waves do not collide is that they only appear to us to across the same region of space or the same dimension (three dimensions for the physical universe), whereas, in reality, this is not the case. The medium in which these waves exist might consist of more than three dimensions (thought of as infinite dimension or spacetime, i.e., infinite or limitless wavy energy). However, humans have evolved to live with organs of vision that allow us to only view things in three dimensions. The next question then becomes the following: “How can a medium with more than three dimensions exist, while the universe that we observe through our eyes is only made up of three dimensions?” This is because what we observe as humans, using the sense organ that we know as our eye, is not a complete, correct representation of the environment. What we observe is a projected reality, projected energy, or ‘limited part’ of reality (waves/energy/dimension).

The limited projection of reality (energy) is due to the limited number of waves projected onto our physical universe [22]. Because of limited projection, these waves may appear as intersected (Figure 2A), whereas, in reality, they are not and instead lie in an extra dimension (limitless or infinite wavy energy that has infinite dimension or limitless spacetime). Its interesting element is one that really intersects our physical universe, which was initially formed by a sort of early light vortex (Figure 2B). In relation to this, the waves that intersect our finite early light vortices (or the lowest light) may appear as physical, material, or electrical vortices (a physical universe that follows the laws of physics) (Figure 2C). Therefore, the formed wave-vortices should cover the electromagnetic spectrum or the lowest light (low-dimensional, diffused, and faster vortex), electricity (high-dimensional, directional, and slower vortex), and anything in between with infinite shapes such as material vortices (vortex of matter), which are commonly observed in nature (Figure 2D). Figure 3 summarizes the wavy finite universe, which has a combination of low-dimensional (light) and high-dimensional (electromagnetic/electric/magnetic) wave fields (see later, where it is proposed that the brain might also have both fields or both low and high entropy). 

In summary, this intersection (i.e., infinite waves with finite waves or higher light with lower light) has apparently created an illusion of physical materials (hologram); they are actually waves (their reality or fundamental is energy) but appear as matter (vortex of matter), light, and electric vortices. This idea may explain why atoms or light existing in our physical universe can exist either in particle (matter is illusion) or wave form (reality or origin), i.e., a duality feature. Hence, one may conclude that an illusion of physical material is created by (a) the limited projection of waves and (b) the limited consciousness of the brain. As mentioned before, limitless consciousness exists as one entity, i.e., one consciousness (wholeness). Therefore, it also encompasses our brain consciousness. According to this argument, one may, hence, view our brain consciousness as not truly representing one whole consciousness. It is only partial or limited. Therefore, one may say that the human brain has limited consciousness. Similar to the ‘limited projected waves’ concept that was previously discussed, one may notice that the word ‘limited’ again appears in our discussion. Thus, with this limited consciousness concept that exists in our brain, we gain a glimpse of the original wave function of particles when collapsed. Thus, one cannot see the original waves (or energy); what we instead see are illusionary vortices of the limited projected waves. Since our interpretation of reality is represented by continuous waves or quantum energy (not particles or matter), our notion is that ‘discreteness arises from continuousness when observed by the mind (brain)’.

## 5. Fusion of Electrical and Quantum Fields inside the Brain: Fusion of Bohmian and Quantum Mechanics and Their Algorithms

Since the brain is also encompassed by the oneness of consciousness (infinite wavy energy), one may presume that the brain features infinite waves. It is delocalized and in diffusion with external cosmos waves. In fact, it can be viewed as one medium of oneness, one field, wholeness, or one consciousness [14,19,20,21]. This indirectly compels us to realize that the brain, in fact, has quantum and multidimensional light features (nonlocal, quantum wave function, diffused, low-dimensional, tunneling, superposition, etc.). In other words, one may view these ‘quantum–light waves of the brain’ (i.e., energy of brain anatomy viewed on the smallest scale) as brain background waves interconnecting and permeating the whole universe, hidden underneath another wavy brain network, i.e., electrical (electromagnetic) brainwaves (functional brain energy). Thus, the brain has two types of energy: (a) anatomical energy (quantum view: anatomy or mass is also considered as energy), and (b) physiological energy (electromagnetic brainwaves). The quantum anatomical energy is hidden and has not yet been detected.

**Figure 4:**
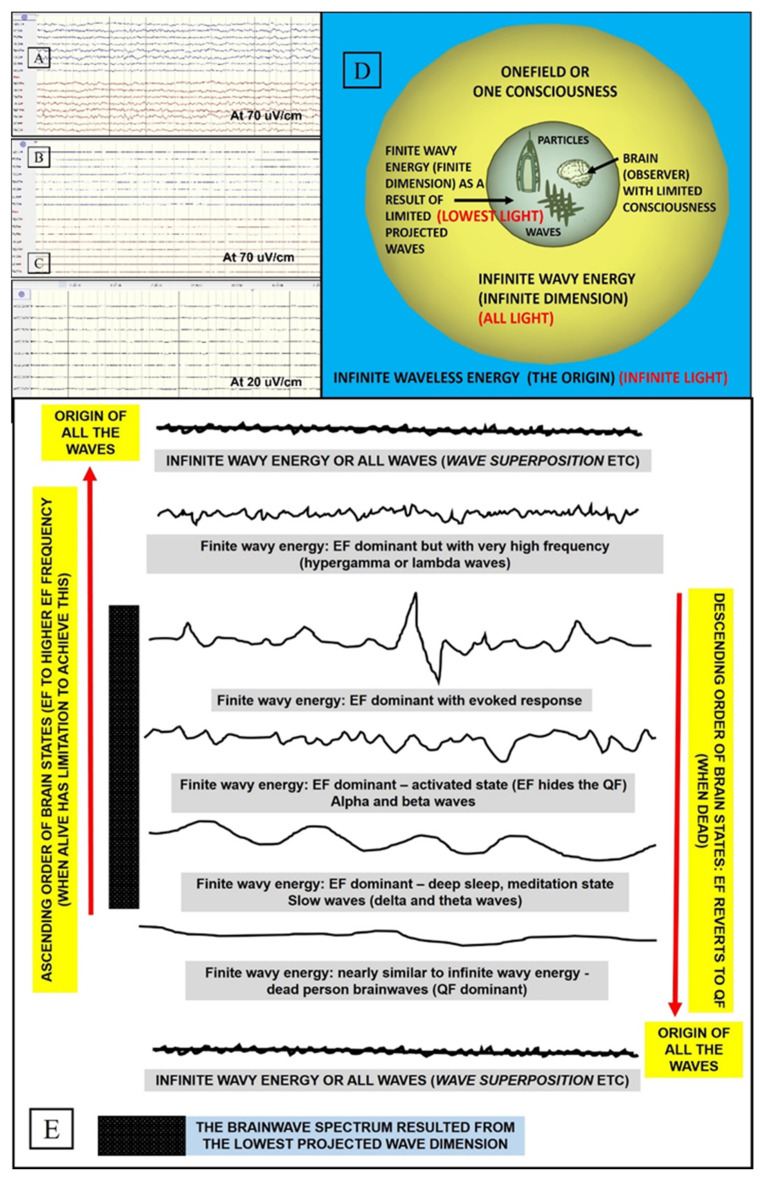
Electromagnetic field, quantum field, and the two ends of the spectrum. (**A**) Scalp electroencephalography (EEG) brainwaves of a healthy person with wavy feature. (**B**) Scalp EEG brainwaves of a dead person at the same sensitivity (70 uV/cm), which now appear isoelectric (flattened). (**C**) Wavy background section of a dead person’s brainwaves recorded directly on the brain surface and viewed at the highest scale (electrocorticography or ECoG at 20 uV/cm). (**D**) The relationship among infinite waveless energy (infinite light), infinite wavy energy (all light/first created light), and finite wavy energy (lowest light). (**E**) The descending order of brainwaves, from electromagnetic field (EF)-dominant to brain quantum field-dominant (dead person), and the ascending order of brainwaves, to the highest EF frequency (hypergamma and lambda) (alive person), may also come closer to infinite wavy energy, oneness, or one consciousness (heightened state of consciousness).

The electrical wave brain network (functional brain energy) is commonly studied and is easily evoked by physical stimuli. Projected waves (limited waves) or vortices which form physical stimuli cause evoked responses inside the brain, which is theorized here as having only limited consciousness (i.e., limited representation of true waves); furthermore, the brain itself is a part of the projected wave-vortices. Thus, one may say that the ‘electrical wave brain network’ represents a kind of mirror to ‘limited projected waves’ (finite physical universe items being compressed into the brain). With this view, i.e., considering the presence of limitations in both wave projection and consciousness, larger evoked electromagnetic fields are naturally observed for seeing, hearing, tasting, etc., of material objects [23,24]. At this juncture, we would like to consider the brainwaves of a dead person according to our clinical observation and our recently published manuscript [25]. It is commonly stated that the brainwaves of a dead person are isoelectric or flattened; however, in most circumstances, one might notice a slight wavy background, which may suggest that tiny background quantum–light waves (energy of the brain anatomy viewed on the smallest scale) could indeed be present. This wavy background is, however, only detectable using direct brain electrocorticography (ECoG) on the surface of the brain (Figure 4A–C). One of the scientific explanations in this regard might be related to our limited consciousness concept. The electrical field featuring very wavy electrical brainwaves, which is dominant when alive (i.e., the brain constantly reacts to the projected waves), has now reverted to one consciousness where the quantum–light field is dominant (substantially less wavy, nearly flattened, and diffused waves). In other words, the brainwaves are now similar to oneness or one-field waves (i.e., infinite wavy energy) (Figure 4D,E). These arguments may explain why the commonly regarded isoelectric brainwaves (when dead) (downward shift in Figure 4E) and the highest-frequency waves (when still alive) (upward shift in Figure 4E), such as gamma and lambda brainwaves, are both related to a heightened state of consciousness [26,27]. Simply put, these two states are related to the higher energy transition (a shift to the higher dimension or infinite wavy energy, i.e., beyond the ordinary range of the spectrum): one state when still alive and another when dead. Nonetheless, one must bear in mind that attaining and maintaining such high- or low-frequency waves when still alive (even through meditation) may not be an easy task for us. A final note on this part of the argument is that our current universe dimension is associated with the lowest brain frequency bands and, thus, can be regarded as the lowest dimension (Figure 4E).

The subsequent question one might ponder is the following: “Is there any interaction between the electrical wave field (physiological energy) and quantum–light wave field (anatomical energy) inside the brain?” In our opinion, the interaction between these two fields is always present because they originate from the same infinite wavy field, which is diverted into two arms (projected waves/electrical wave field in the brain and quantum–light wave field/quantum level of brain anatomical waves or energy) and finally converges in the brain. This interaction may cause various states. The electrical wave field (evoked fields) dominates with respect to our five senses that are related to matter or projected fields (physical stimuli), whereas a possibly larger role is played by the quantum–light wave field in cognitive processes such as language, memory, attention, emotion, and anxiety (which are not directly related to material stimuli). A disturbed interaction, disharmony, or chaos (decoherence) between these two fields, as well as a reduction or increment in quantum neuroanatomical waves/energy (i.e., alteration in brain mass), may lead to various diseases, as seen in some psychiatric disorders where an ‘exit from reality’ or ‘break in wholeness’ (fragmented reality, fragmented energy, fragmented waves, or fragmented story/description) is commonly present [28,29,30,31,32]. The ‘level of this interaction’ may also explain why one healthy person has a higher anxiety level than another healthy person. Therefore, it is deemed appropriate to carry out electroencephalography (EEG) and magnetoencephalography (MEG) studies; in the future, with the availability of advanced technology (perhaps a sort of quantum wave detector technology, much more sensitive than MEG), one can include quantum field, quantum–light field, or quantum neuroanatomical energy measurements in these psychiatric or neurological patients. Furthermore, one may also want to use some sort of light (light therapy) and/or electromagnetic field (electroconvulsive therapy (ECT) or transcranial magnetic stimulation (TMS)) as a method of therapy for psychotic spectrum disorder or for cognitively impaired individuals. 

**Figure 5:**
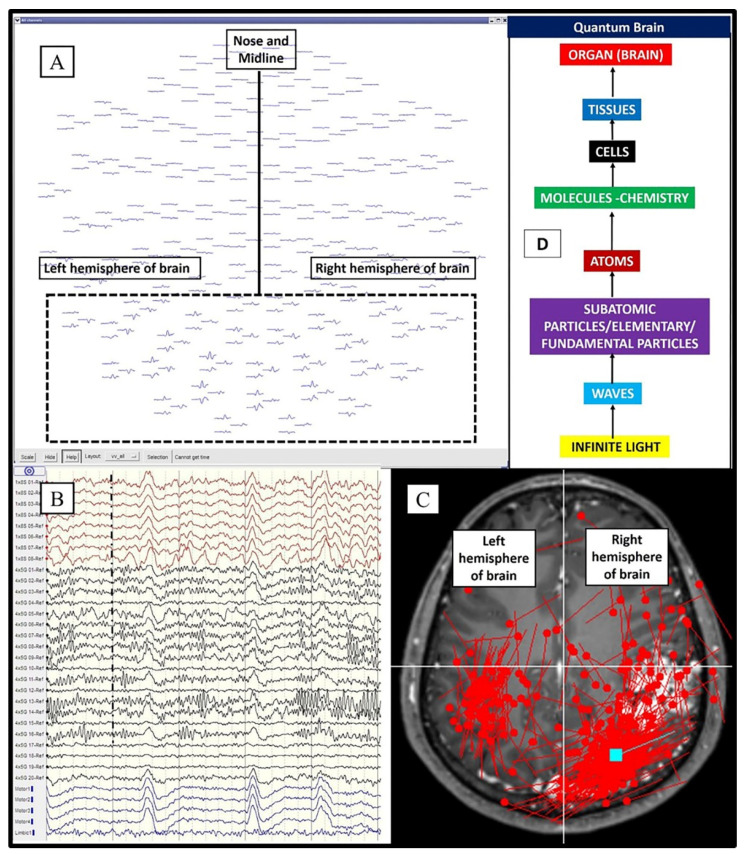
Quantum principles inside the brain. (**A**) The visually evoked magnetic field obtained through magnetoencephalography (MEG) as an example of an entangled electromagnetic field that has bihemispheric inversed potential responses. (**B**) Brainwaves obtained via ECoG (uppermost and middle, i.e., red and black waves) and EEG (bottom, blue waves): the normal areas of the brain (red and blue) have coherence or harmony waves, whilst the black (abnormal) areas exhibit decoherence (chaos/disharmony) in a psychotic epilepsy patient. (**C**) Despite the presence of a lesion in only the right hemisphere of the brain, epileptic spikes detected using MEG were noted in both hemispheres (may also be a sign of entanglement and/or tunneling; note that the slower detectable time response in the opposite brain hemisphere may be due to the measurement instrument used). (**D**) The brain and its anatomical hierarchy; the quantum part remains unexplored.

In a nutshell, both electrical and quantum–light fields are needed to sufficiently study brain function. In other words, one may say that the brain has both an electromagnetic field (EMF) or electric field (EF) and a quantum field (QF) or quantum–light field (QLF); alternatively, it can be said that the brain has both high-EF and low-QF waves. Table 1 illustrates our points which differentiate the brain EF from brain QF, whilst Figure 5 shows the possible existence of quantum principles (entanglement, tunneling, and coherence) inside our brain. In physics, the concept of these two brain networks can be explained using Bohmian and quantum mechanics [33]. For quantum or background quantum–light brainwaves (difficult to locate and diffused), a nondeterministic algorithm should be used to study them, whereas, for electrical brainwaves (with features of a pilot wave, locatable, directional, and deterministic with a specific network), a Bohmian mechanics algorithm should be used to study them. For the fused brain field (EF + QF), a combined Bohmian and quantum algorithm may be the answer. As a final point, it is worth noting here that the brain is the only human anatomical structure (not bone, kidney, muscle, heart, etc.) which is seen as capable of interacting meaningfully with the cosmos quantum field. The total interaction of the brain QF (anatomical QF) with the cosmos QF, as well as with the brain electric field (EF), results in the formation of the ‘total brain field’, or perhaps an alternative name for it is “the mind field”. The reason for the brain being the only organ capable of interacting meaningfully with the cosmos QF may lie in ‘the seat of the soul’ brain concept (deep brain area that converges all waves), which has been debated since ancient times and was proposed by Leonardo da Vinci as being situated at the center of the brain. Thus, this deep and central part of the brain seems to be a mysterious and fascinating brain region that deals with everything from human consciousness to the determination of being alive or dead and awake or asleep, in addition to being associated with all brain–body functions [34,35]. In line with these arguments, the mushy brain should not be regarded as an organ and, thus, cannot be transplanted.

## 6. Conclusions

**Figure 6:**
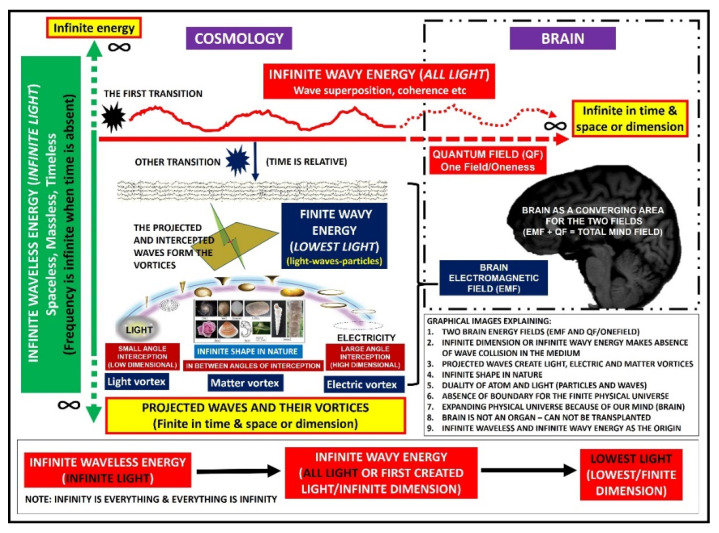
A graphical summary of our new perspective on brain function, which covers infinite waveless (infinite light), infinite wavy (all light), and finite wavy energy (comprises the lowest light), projected wave-vortices, quantum field, and electromagnetic field.

According to our new perspective on brain function, infinite waveless energy is an infinite frequency (energy) that is not related to time or space. It gave rise to another infinite energy, called infinite wavy energy, which is related to infinite space–time or dimension. Since time is only relative, new finite wavy energy (our universe) with a ‘new period of time’ can be formed via a second transition. The formation of new finite wavy energy/physical universe (started with the light) has resulted in the subsequent intersection of various waves coming from higher infinite wavy energy/waves/dimension. These intersections manifest themselves as various infinite-shape vortices that follow the laws of biology and physics. These vortices range from light to electricity. Matter and other vortices are included in between. Therefore, matter can be regarded as an illusion or can appear as either particles or waves (duality). This concept also explains our physical universe (finite wavy energy) as having no border. In reality, it is part of an infinite wavy energy/universe and, thus, the noted physical universe expansion could in fact be a consequence of our brain activity, which has partial consciousness (always sees waves or energy as particles). In essence, a person with limited brain consciousness cannot reach the border (no border or no question as to what substance the universe expanding into) of this illusionary universe (the brain itself is regarded as a projected vortex, which shall always notice the expansion of finite and angulated projected waves or the illusionary universe). According to these principles, our brain is thought of as having two types of brainwaves or energy: (1) quantum–light waves, which permeate and interact with the whole universe (one field/oneness/infinite wavy energy, related to macro and micro brain anatomical energy or brain particles which are viewed as waves, in tally with the string theory of the universe), and (2) the electromagnetic field or electric field (related more to brain physiological energy), which corresponds to a limited or finite projected wave (finite wavy energy) with a larger evoked response when stimulated. Thus, the brain can be regarded as an incomplete mirror to our cosmos (limited projected wave-vortices resulting in EMF and part of a more diffused QF). This manuscript ends with Figure 6 summarizing our whole perspective on this interesting aspect of brain function, which is closely related to cosmology.

## Figures and Tables

**Table 1:** General features of the brain electromagnetic field (EF or EMF) and brain quantum field (QF) or brain quantum–light field (QLF).

	Feature	Electromagnetic or Electric Field (EMF)	Quantum Field (QF)
1	Origin	Projected waves (physiological waves)	Original waves (anatomic–chemical waves)
2	Wave pattern	Presence of pilot/directional wave	Diffused (delocalized) waves
3	Wave characteristics	High-frequency wave (limited frequency range when alive)	Low-frequency wave (unlimited and covers the whole frequency range via quantum wave superposition)
4	Wavelength	Short wavelength (presence or absence of wave superposition)	Long wavelength (with absolute presence of wave superposition)
5	Quantum concept	Deterministic (locatable)	Nondeterministic (unlocatable, changing)
6	Physics concept	Bohmian mechanics	(Truly) quantum mechanics
7	Dimension	High dimension (electric)	Low dimension (light)
8	System (energy) Note: The brain is like a mirror to the cosmos, with the brain and universe both having “low and high entropy”. Low entropy is formed from high entropy.	Concentrated organized system (energy) (low entropy) Low entropy is formed from the high-entropy state (EMF/EF originates from the projected and intersected QF waves)	Widespread diffused random system (energy) (high entropy) High entropy gives rise to the low-entropy state (the projected and intersected QF waves which form various vortices give rise to EMF/EF)
9	Brain network	Simple or specific network (few nodes)	Complex, changing, or nonspecific network
10	Symmetry	More asymmetry	More symmetry
11	Evoked response	Large evoked response with few stimuli or trials	Smaller evoked response, which needs a greater number of stimuli/trials and a quantum wave detector
12	Neuroplasticity (recovery once treated)	Less likely (because of limited field or focal/less diffused waves)	More likely (because of diffused waves and oneness)
13	Wholeness/oneness/one field concept	No (it is a response to the projected/limited field)	Yes (spreading, interconnecting, and permeating entire universe/field)
14	Related to psychiatry	Less relevant	More relevant (the brain QF is thought of as related to wholeness, reality, or the one consciousness concept)
15	Medical concept	Currently described by the Newtonian concept, whereby the brain is divided into anatomy/ensemble of particles and waves (physiological or electromagnetic brainwaves, i.e., the commonly studied waves)	Proposed as the medico-quantum physics concept, whereby an anatomic–chemical quantum brain field exists which remains hidden (anatomy/particle is also regarded as energy quanta/waves or mass is also regarded as energy) When this diffused field combines with the brain electric field, we get the total brain field ^#^
16	Why is only the brain involved in interacting meaningfully with these two fields? Is it related to the seat of human soul concept?	The brain is the only human structure where these two energy fields (pairs) “converge”, Likely (because the center part of the brain is the origin for physiological brainwaves/EMF or all body functions (seat of soul))	The brain is the only human structure where these two energy fields (pairs) “converge” Likely, the cosmos QF can interact meaningfully *with only the brain*, which has a seat of soul concept, resulting in the *total brain field or mind field (see # above)*
	**Brain Function**	**Combination of EMF and QF (Total Brain Field or Two Brain Fields/Energy)**	
A	Brain function (motor, sensory, vision, sound, touch) and its impairment	Noncognitive impairment such as stroke affecting motor, sensory, vision, sound, touch. EMF is more affected than QF. It is measurable and associated with *degree of impairment*	
B	Brain function (language, emotion, memory, attention, planning, etc.) and its impairment	Cognitive impairment of language, emotion, memory, attention, planning, etc. QF is affected significantly, together with EMF. It is also measurable and associated with *degree of impairment*	
C	Brain function and psychosis	Psychotic manifestations such as auditory or visual hallucinations, thought insertions, and delusions QF is likely more affected than EMF (i.e., not related to projected waves or, in other words, no stimuli) or there is an abnormal total brain field or abnormal interaction between the two fields -Yes or no; *presence or absence, i.e., not associated with degree of impairment)*	

## Data Availability

The data presented in this study are available on request from the corresponding author. The data are not publicly available due to our hospital data privacy.
